# An Intelligent Space for Mobile Robot Localization Using a Multi-Camera System

**DOI:** 10.3390/s140815039

**Published:** 2014-08-15

**Authors:** Mariana. Rampinelli, Vitor Buback. Covre, Felippe Mendonça. de Queiroz, Raquel Frizera. Vassallo, Teodiano Freire. Bastos-Filho, Manuel. Mazo

**Affiliations:** 1 Department of Electro-Mechanics, Federal Institute of Education, Science and Technology of Espirito Santo (IFES), Estrada da Tartaruga, s/n, 29215-090, Guarapari, Espirito Santo, Brazil; 2 Department of Electrical Engineering, Federal University of Espirito Santo (UFES), Av. Fernando Ferrari, s/n, 29075-910, Vitoria, Espirito Santo, Brazil;E-Mails: vitorcovre@gmail.com (V.B.C.); mendonca.felippe@gmail.com (F.M.Q.); raquel@ele.ufes.br (R.F.V.); teodiano.bastos@ufes.br (T.F.B.-F.); 3 Electronics Department, University of Alcala, Campus Universitario s/n, 28805, Alcala de Henare; Madrid, Spain; E-Mail: mazo@depeca.uah.es

**Keywords:** intelligent space, multi-camera network, mobile robot localization

## Abstract

This paper describes an intelligent space, whose objective is to localize and control robots or robotic wheelchairs to help people. Such an intelligent space has 11 cameras distributed in two laboratories and a corridor. The cameras are fixed in the environment, and image capturing is done synchronously. The system was programmed as a client/server with TCP/IP connections, and a communication protocol was defined. The client coordinates the activities inside the intelligent space, and the servers provide the information needed for that. Once the cameras are used for localization, they have to be properly calibrated. Therefore, a calibration method for a multi-camera network is also proposed in this paper. A robot is used to move a calibration pattern throughout the field of view of the cameras. Then, the captured images and the robot odometry are used for calibration. As a result, the proposed algorithm provides a solution for multi-camera calibration and robot localization at the same time. The intelligent space and the calibration method were evaluated under different scenarios using computer simulations and real experiments. The results demonstrate the proper functioning of the intelligent space and validate the multi-camera calibration method, which also improves robot localization.

## 1. Introduction

In 1969, in the novel, Ubik [1], Philip K. Dick described a society able to interact with smart objects and environments in a natural way, through speech and gestures. Inspired by this novel, in 1998, Weiser speculated that the technology of the future would be so immersed in people's lives that it would be unnoticeable [2]. Back when the mainframe started to be put aside, and most research was directed to developing a personal computer, Weiser stated that it was not the ideal tool yet. In his opinion, a good tool would be invisible, *i.e.*, the user's attention should be focused on solving the problem, not on the tool itself [3]. As such, computing should be used unnoticed and without the need of technical knowledge about the equipment, which still happens to date in personal computers and several electronic devices. By this definition, Weiser is considered the father of ubiquitous computing.

The core concept in ubiquitous computing is that computing is to be immersed in an environment occupied by people, allowing interaction in a natural way, through gestures and speech [2], providing users services that improve their quality of life.

Studies about intelligent spaces have arisen from research in ubiquitous computing applied to immersing computing in the environment as a whole. Intelligent spaces can be described as an environment equipped with a network of sensors (for example cameras, microphones, ultrasound) able to gather information about the surrounding world and a network of actuators (robots, personalized wheelchairs, information screens) that enables user interaction and task execution. Besides that, both sensors and actuators are subject to a supervisor system, able to analyze the information from the sensors and make decisions [4].

Research on intelligent spaces can be divided into the ones dedicated to corporate environment and domestic usage. Projects in the corporate environment aim to ease people localization in corporations, communication among coworkers and to personalize the workplace, as seen for Active Badge, in Cambridge [5], Intelligent Room [6] and Smart Room [7], both at MIT.

On the other hand, studies in domestic contexts, in general, intend to improve the residents' quality of life and reduce costs with power, water and other supplies. Examples include EasyLiving from Microsoft [8]; Adaptive House from the University of Colorado [9]; Aware Home from the Georgia Institute of Technology [10]; and MavHome [11,12] from Washington State University and the University of Texas at Arlington. Despite automating routine activities in an intelligent way, the aforementioned intelligent spaces do not incorporate yet the usage of mobile robots as actuators.

However, there are already some projects that join the concept of intelligent spaces and mobile robots, such as Intelligent Space [13] from the University of Tokyo, MEPHISTO (Modular and Extensible Path Planning System Using Observation) [14] from the University of Karlsruhe and the ISPACE (Intelligent Space) from the University of Acala [15], where robots may receive commands from the environment. However, even in these cases, the intelligent spaces are indoor environments, mainly domestic.

Considering larger environments, there are systems that are being developed using networked sensors and robots with ubiquitous intelligence. The Japan NRS (Network Robot System) Project used four robots in real environments: a museum and a shopping mall [16,17]. Additionally, research focused on outdoor environments was developed by the URUS (Ubiquitous Networking Robotics in Urban Sites) Project, involving 11 institutes and tests environments at different universities in Europe [18]. Furthermore, the work done in [19,20] proposes a system with intelligent cameras and autonomous robots, aiming at system scalability.

Among the several tasks of an intelligent space, it may be cited the guidance of one or more mobile robots or robotic wheelchairs and gesture recognition. Smart houses are able to adequate ambient temperature and light, detect residents with disability or an elderly person falling and communicate this to an emergency system to improve the residents' comfort. Therefore, intelligent space applications are numerous and involve many fields in service robotics, security tasks, automatic vigilance and people with disabilities assistance.

In this context, this work presents the intelligent space built at the Federal University of Espirito Santo (UFES). Specifically, the intelligent space described in this paper aims to achieve localization, identification and control of mobile robots or robotic wheelchairs. In [21], a robot navigation control strategy using a network of cameras was proposed and tested in a 45-m^2^ laboratory, demonstrating the viability of the usage of such a sensor in the control of mobile robots.

In order to make a mobile robot or wheelchair follow a desired path, the control system must obtain information about the location of the robot itself and its environment. Instead of having many sensors, actuators and computational power onboard the robot, the most complex part in the sensorial and processing structure is transferred to the environment, simplifying the robot's structure and reducing costs. Using this approach, to guide wheelchairs indoors is interesting, once intelligent spaces can offer other tasks to improve the quality of life of its users, such as identifying risk situations. Other than that, intelligent spaces allow more than one mobile robot to be guided simultaneously without the need to increase the existing infrastructure complexity.

The correct location and identification of every element in the environment is a fundamental task for intelligent spaces, especially if the environment controls mobile robots. Academic and industrial research have created location devices that differ concerning precision, reach, updating speed, maintenance and installing costs [22].

In outdoor environments, GPS and cell phone devices have reached good levels of precision, achieving centimeter-level precision in some cases. However, the localization problem in indoor environments is still subject to much discussion [22,23]. Some of the technologies used for indoor localization achieve centimeter-level precision, such as ultra-wide band (UWB) [24], ultrasound [25], infrared [26] or computer vision [21].

From the mentioned technologies, the usage of computer vision in intelligent spaces is one of the most promising, due to its capacity to provide a large amount of information about objects and users in the workplace and the controlled robotic vehicles [27,28]. Besides that, the field of view of a single camera is relatively large when compared to other sensors. The usage of cameras as sensors in intelligent spaces is not limited to mobile robots localization, but also to object and gesture recognition, as much as risk situations, such as falling. At last, cameras are already present in many environments, such as commercial centers, hospitals and schools, and could be used as the sensors for the intelligent space. Therefore, the sensors chosen to be used in the intelligent space developed in UFES were video cameras.

However, to allow identification and localization of mobile robots, robotic wheelchairs, persons or any other object in the intelligent space, it is necessary to know correctly the location and internal characteristics of each camera. Such information is obtained through a calibration process, which provides an estimation of the parameters that define the projection of 3D points in space to 2D points in the image [29,30]. The precision obtained in localizing and tracking objects in the intelligent space depends directly on how precise the calibration of the cameras is. In general, calibrating a camera is an exhaustive task to do manually, which becomes worse with the increasing number of cameras. Besides, it is not a trivial task.

Taking that into consideration, an automatic camera calibration method is also proposed in this work. It is used to calibrate the cameras in the intelligent space at UFES and will be also described in this paper. In order to make this process more automated and less costly, as is usual in different systems [31,32], the described algorithm simultaneously performs the calibration of the multi-camera network and the localization of a mobile robot. Differently from other works presented in the literature, in the proposed algorithm, no characteristic of the cameras is known *a priori*, and these sensors are located close to the ceiling, as would happen in a real situation. As a result, the calibration of the multi-camera network and robot localization are done simultaneously.

Summarizing, the objective of this paper is to describe the intelligent space built at UFES and to present the calibration process of the multi-camera network. The intelligent space structure is similar to ISPACE [15] at the University of Alcala (Spain), a cooperation partner of UFES, but specific hardware and software were developed locally to allow the proper communication between all of the sensors nodes in the network. Besides that, an automatic calibration process for all of the cameras was proposed and implemented, reducing the need for human intervention.

This paper is organized as follows: The next section describes the intelligent space developed in our laboratory. Section 3 describes the mathematical model of the mobile robot and the cameras. Section 4 presents the algorithm for calibration of the multi-camera network and robot localization. Section 5 presents the results achieved with the proposed algorithm, both in simulations and in real experiments. Finally, Section 6 discusses the results and suggests future work.

## 2. The Intelligent Space

**Figure 1.**
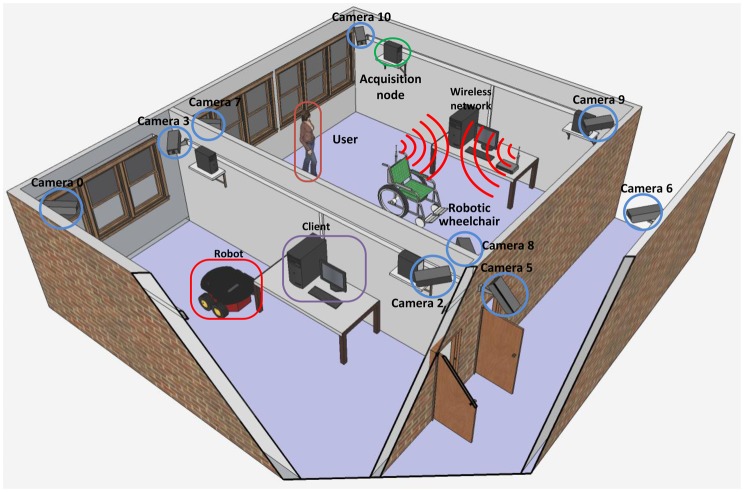
Three-dimensional structure of the intelligent space assembled at the Federal University of Espirito Santo (UFES).

The intelligent space built at UFES occupies two laboratories, measuring 62 m^2^ and 40 m^2^, and the corridor between them. Implementing this intelligent space required developing a system to do image acquisition from the cameras placed in the laboratories and the corridor, to perform image processing in order to extract information from images and to establish communication among the computers that are attached to each camera of the network. It is worth mentioning that the image acquisition must be properly synchronized among the cameras. Thus, to compound the intelligent space, a client/server architecture was implemented, which allows image acquisition and processing, while balancing the processing load required among the servers. A 3D model of this structure can be seen in Figure 1.

### 2.1. Hardware Architecture

As previously mentioned, the sensing system chosen for this intelligent space is a set of synchronized cameras, *i.e.*, images from all cameras are captured at the same time. Altogether, 11 cameras are used, with four cameras in each lab and three cameras in the corridor. The cameras model is DBK 21AUC03 from The Imaging Source [33], with a USB interface and a 640 × 480 pixels color CMOS sensor, with 76 frames per second (fps).

Each camera is connected via an USB interface to a processing node able to capture images, process them and send the results to another computer. The acquisition and processing node are single-core 1.8 GHz CPUs with 1 GB of RAM. The decision of having USB cameras connected to computers instead of IP cameras was made, because we wanted to have distributed processing among the network nodes, without overloading the network with an intense data transmission. Besides that, the centralizing computer, which must coordinate the tasks inside the intelligent space, receives just the essential data needed for that. Thus, with a lesser volume of processing, the central node will be able to coordinate more than one task simultaneously.

**Figure 2.**
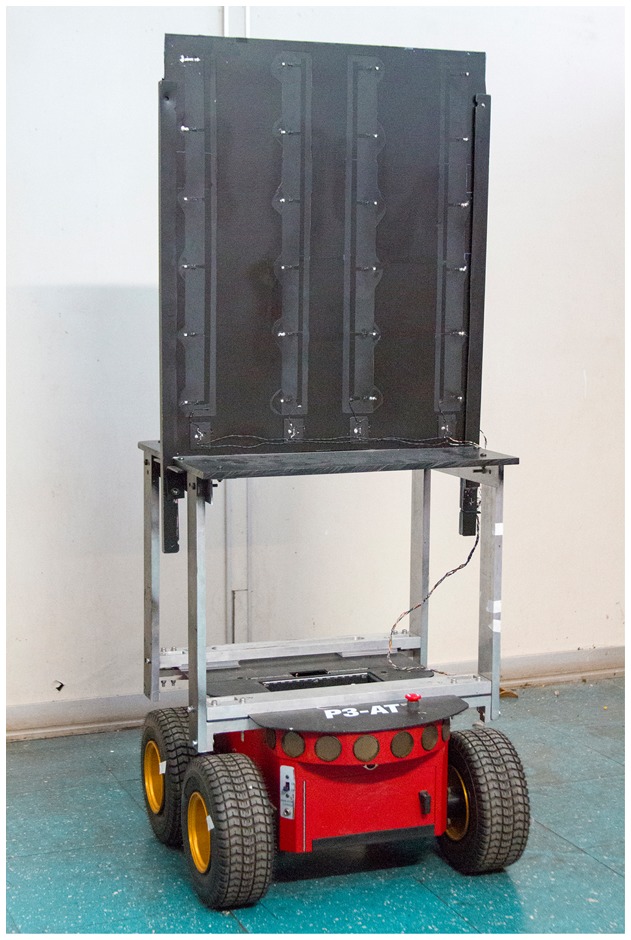
Pioneer 3 AT used in the experiments in the intelligent space at UFES.

The robot used in this work is a Pioneer 3 AT (P3-AT) from Adept MobileRobots [34]. The P3-AT is a four-wheel drive robotic platform, weighing 12 kg and measuring 27.7 × 49.7 × 50.8 cm with encoders and sensors, as shown in Figure 2. The robot is equipped with an onboard computer with a wireless IEEE 802.11 communication interface in order to receive control information and to send data from its sensors.

**Figure 3.**
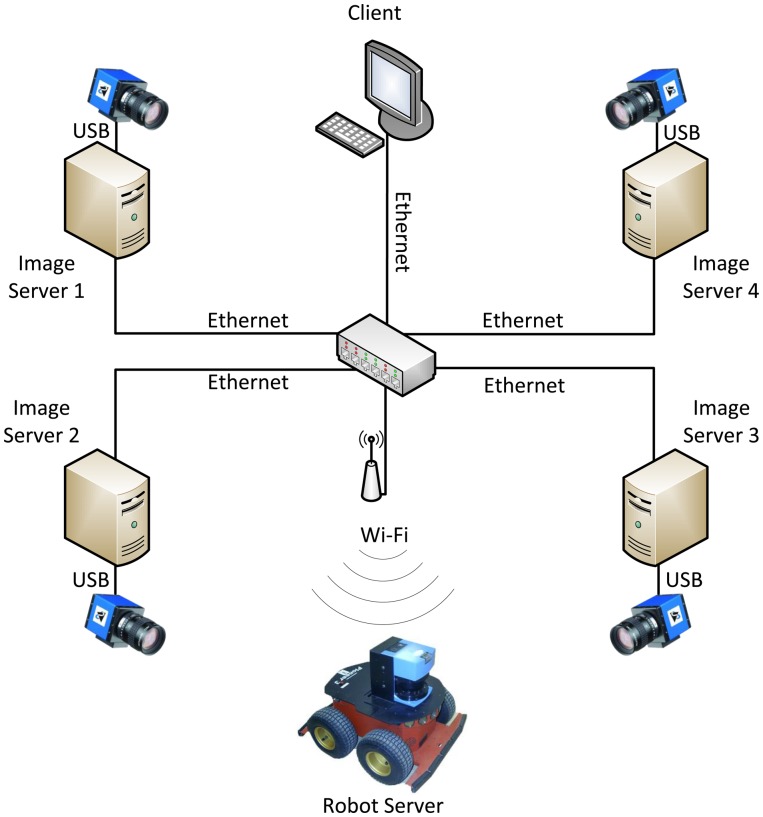
Diagram of hardware/software architecture in the intelligent space at UFES.

The processing nodes are interconnected via a switch in a LAN (local area network) for a gigabit Ethernet interface, using a star topology. Besides, a wireless router was added to the network to allow the robot to connect to the system. A simplified schematic of hardware and software structure representing four cameras of one of the intelligent space's laboratories is shown in Figure 3.

### 2.2. Software Architecture

**Figure 4.**
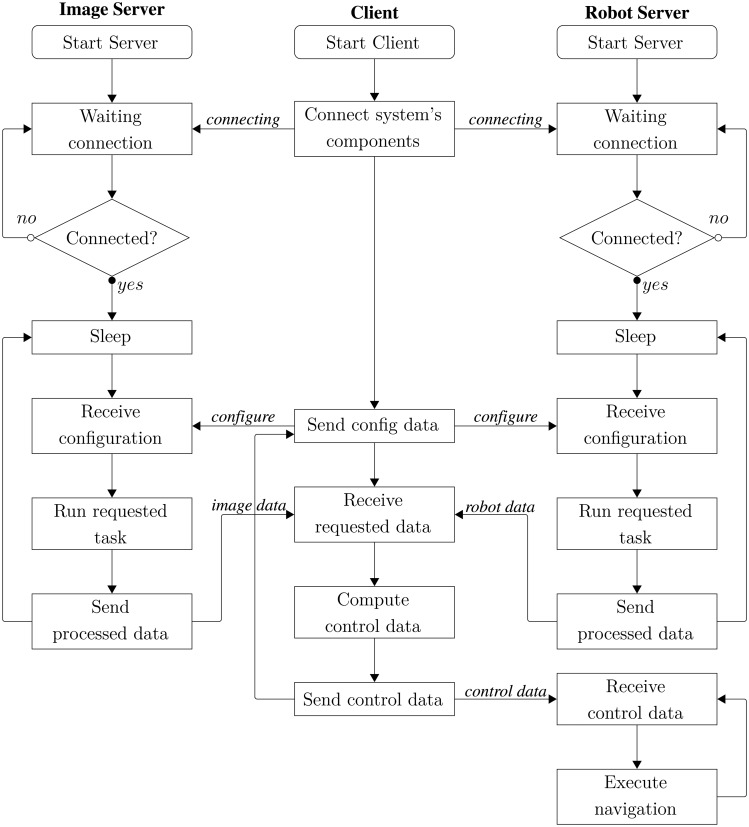
Flowchart of the basic structure of the intelligent space.

The programming architecture used in this system is a client/server with TCP/IP connections. Communication is established when the client sends a request to the server through the network. The server receives the message, executes the request and sends a response back to the client. The basic structure of the intelligent space software is presented as a flowchart in Figure 4.

In the intelligent space, each camera is connected to only one acquisition and processing node, the image server, which is connected by a switch to a central computer, the client. Each processing node has a fixed network address (IP), and thus, it is possible to recognize with which camera the system is working. The main task of the image server is to capture images from its camera, store them if requested, perform low-level image processing and to send the obtained data to the client. The processing performed by the image server provides information needed for executing the robot's activities based on, e.g., image segmentation, artificial marks identification and filtering.

The image servers perform the largest volume of processing in this system, since each analyzed frame has a large amount of information. For instance, an image captured by a camera attached to a server has 307,200 pixels, and each pixel has three bytes of data. Thus, when an image server captures and processes 15 fps, that means it processes approximately 13 MB/s.

Some libraries were used to develop the image server system, such as OpenCV library [35], which allows a fast and high-level image processing; winsockets API (application programming interface), which allows sending and receiving data over the network without any programmer concern with the network layer below the application layer, and the pthread library, which allows the image processing to occur in parallel with the data transfer through the network.

The client function is to request information from the servers, to centralize the data provided by them and to determine which tasks will be performed in the intelligent space. In the client, the data obtained from the image servers and the robot server are consolidated to be used in any task that requires the collected information. For instance, such tasks can be the 3D reconstruction of the robot's pose or the robot control defined by the user. The amount of data received by the client is small when compared with those processed in the image servers.

The client is the only component of the system with a graphical user interface (GUI). From the GUI, one can perform communication settings with the image server (e.g., define which cameras are connected, the frame rate and image resolution) and with the robot server (e.g., choose maximum linear velocity, maximum angular velocity and enable joystick). Furthermore, the GUI starts the connection to the servers, sends start/end task commands and disconnects the chosen servers. Thus, the whole system is configured and initialized by the client.

The synchronization for capturing images and robot's odometry data is also the client's responsibility, which sends the capture command to all servers at the same time.

The robot server is connected to the robotic platform and is responsible for sending the obtained data from robot's sensors to the client and for receiving motion control commands from the client. The communication between client and robot server should be wireless in order to allow robot motion.

Due to the fact that we are doing preliminary tests with the intelligent space, in this work, only one lab was used for the experiments and for the development of the multi-camera calibration method, which shall be extended to the whole intelligent space as soon as the desired results are obtained. Consequently, in this paper, just four of the 11 cameras of our intelligent space were used.

#### 2.2.1. Communication and Synchronization

In the intelligent space, the communication between the servers and the client occurs in both directions and, depending on the amount and type of the requested information, there would be two or three communication sections. Thus, a lot of information can be sent in parallel in the system.

**Figure 5.**
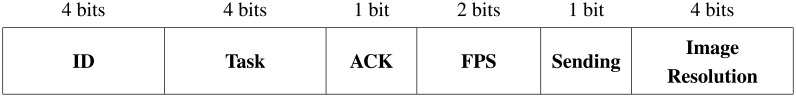
System data block “commands”.

In order to facilitate the communication process, a communication protocol called “commands” was defined, which contains some variables sent between the client and the servers in order to configure what is performed in the system. The data block defined in this work is shown in Figure 5. It contains the ID of who is sending the data (ID), the type of task to be performed (task), a sign to confirm or not that the previous information has been successfully transmitted over the network (ACK), if frames are sent or not for user viewing (sending), the maximum frame rate (FPS) and the resolution of the captured image (image resolution).

Once the communication of the supervisory system was working properly, the synchronization of the intelligent space's components was made. It works like this: the client sends the request for reading data; then, the servers capture the data from all of their sensors at the same time, *i.e.*, camera, encoder and gyroscope; after that, the servers process the data and send the results back to the client.

**Figure 6.**
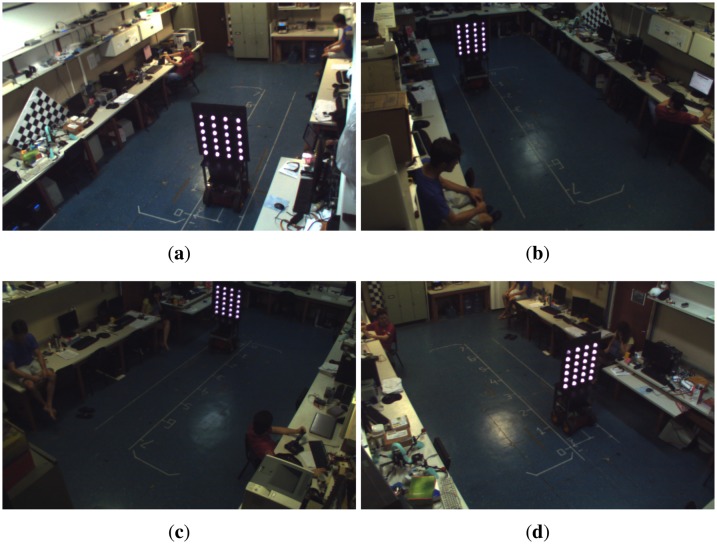
Images captured by the four cameras at the same instant. (**a**) Camera 1; (**b**) Camera 2; (**c**) Camera 3; (**d**) Camera 4.

To verify the effectiveness of the supervisory system synchronization, we conducted a test in which the robot performed a straight path. Throughout the whole experiment, images of the robot were captured using four cameras of the intelligent space. Figure 6 shows the images captured at the same instant.

It can be noticed that in all images, the robot is located at the same spot, which means that the synchronization of all cameras worked well, and thus, the images were captured at the same time.

### 2.3. Algorithm for Marks Detection and Identification

To perform the calibration of the multi-camera network in the intelligent space, a pattern of 50 × 60 cm containing 24 infrared LEDs on both sides was fixed on the top of a robot. Such LEDs are arranged to form a 6 × 4 grid, with each LED distanced 10 cm in the vertical direction and 15 cm in the horizontal direction. Once the intelligent space cameras do not have an infrared filter, the infrared LEDs are seen as a circular white spot, as shown in Figure 6, which makes the initial image segmentation easier. To a proper mark identification, one LED in each face of the pattern is powered with a different electrical current, resulting in reduced brightness, so this mark is used as the pattern's reference.

To identify the 24 marks on each one of the pattern's face, a sequence of operations is applied to the captured frame in order to obtain a binary image where the contours of every object are detected and stored. If the camera can see one of the pattern's faces completely, it is very likely that the contours representing the LEDs marks are among the obtained contours.

Some contours are initially removed based on empirical thresholds, such as minimum and maximum area, minimum number of points that form a contour and non-convexity. For each remaining contour, an approximated ellipse is determined, whose area is calculated from its axis. If the ratio of the contour area to the ellipse area is close to one, it means that the object corresponding to the contour is similar to an ellipse, so it is a candidate to be one of the pattern's marks. Otherwise, these objects are also discarded.

After this classification, if less than 24 marks are detected, it is possible that the calibration pattern is not entirely seen by the camera, and so, the frame is discarded. Otherwise, the barycenter of the detected marks is calculated. The 24 closest marks are, likely, the marks from the calibration pattern. With the possible marks classified, the smallest one (in area) is identified as the reference one and, from such a reference, the remaining marks are sorted.

Once the 24 marks are classified and sorted, it is necessary to check if they actually form the calibration pattern. To perform this task, the distance between neighboring marks in each line is calculated and normalized. Then, for each line, the standard deviation of these distances is calculated. The same is done to the marks in each column. If all of the standard deviation values are smaller than an empirical threshold, then the 24 marks are considered part of the calibration pattern. In the case that at least one of these values is greater than the defined threshold, the frame is discarded, and it is considered that the calibration pattern could not be detected in that frame.

## 3. Mathematical Notation

This paper introduces the intelligent space built at UFES and the automatic calibration method of its multi-camera network. For a better understanding of the algorithm presented in this work, this section describes the mathematical models used to represent the robot and the cameras.

### 3.1. Robot Model

**Figure 7.**
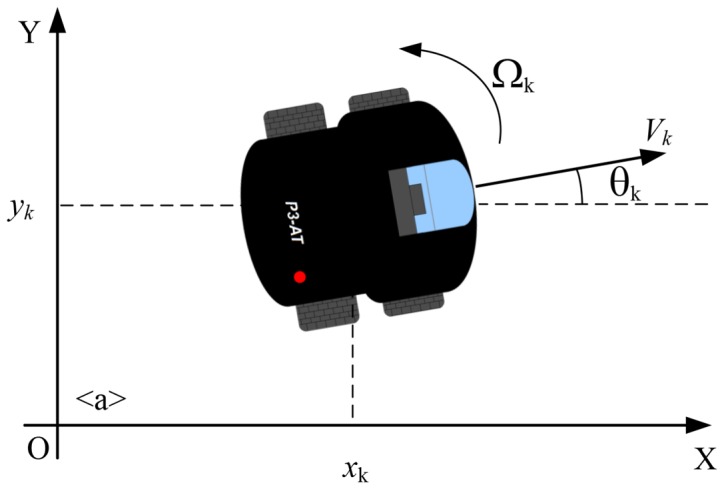
The robot model used in this work.

The robot used in this work is a skid steer platform, shown in Figure 7. Once it uses encoders with inertial correction to compensate for skid steering, the robot can be modeled as a differential drive robot, whose velocity is described as:

(1) $$\left(\begin{array}{c} \dot{x}\\ \dot{y}\\ \dot{\theta } \end{array}\right)=\left(\begin{array}{c} V\text{cos}(\theta )\\ V\text{sin}(\theta )\\ \Omega \end{array}\right)$$

The encoders provide the current position and orientation of the robot, *i.e.*, the robot pose on the ground in relation to an initial vector. This initial vector corresponds to the robot initial pose in the world coordinate frame, *O_w_*. For this work, we focused on an indoor environment, and because of that, robot motion is considered to be constrained to the *xy*-plane. The robot pose at time *k* is represented by **X***_k_* = (*x_k_,y_k_, θ_k_*)*^T^*, where *x_k_*, *y_k_* are the robot position and *θ_k_* its orientation, which corresponds to a rotation around the *z*-axis that is orthogonal to the ground. Without loss of generality, it is considered that the initial pose of the robot matches the origin of the world frame, *O_w_*, with no rotation, *i.e.*, the *x*-axis of the robot coincides with the *x*-axis of the world reference frame.

**Figure 8.**
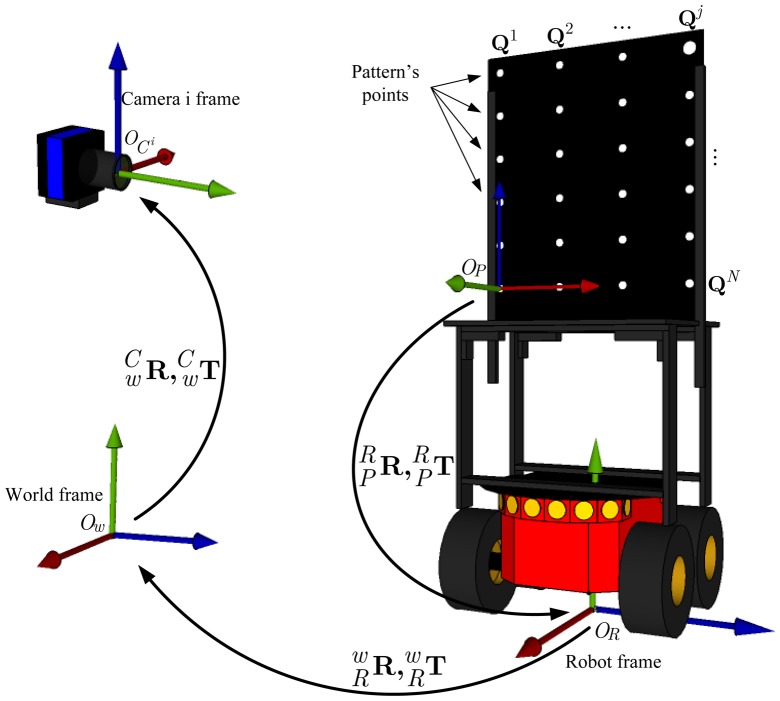
Spatial relation between the world frame *O_w_*, the robot frame *O_R_* and the camera i frame *O_C_*.

Therefore, considering the initial pose of the robot as **X**_0_ = (0,0,0)*^T^*, the encoders capture the sequence of poses **X**_1_, …, **X***_K_* assumed by the robot at the instants of time *k* = 1, …, *K* while it moves along a path. The sequence of poses in the time interval *k* = 1, …, *K* is represented by the vector $\text{X}={\left({\text{X}}_{1}^{T},\ldots ,{\text{X}}_{K}^{T}\right)}^{T}$ Figure 8 shows the spatial relationship between the robot frame, *O_R_*, the world frame, *O_w_*, and the reference frame of one of the cameras, $O_{C}^{i}$, inside the intelligent space.

#### 3.1.1. Robot Motion Model

In this work, the robot motion is modeled by a function **X***_k_* = *g*(**X***_k_*_-1_,**U**_k_), where **X***_k_*_-1_ is the previous pose and **U***_k_* is a vector containing the values of the linear and angular velocities provided by the encoders. Based on Equation (1), the model for the robot movement is defined as:

(2) $$\left(\begin{array}{c} x_{k}\\ y_{k}\\ \theta_{k} \end{array}\right)=\left(\begin{array}{c} x_{k-1}+V_{k}dt\text{cos}(\theta_{k-1}+\Omega_{k}dt)\\ y_{k-1}+V_{k}dt\text{sin}(\theta_{k-1}+\Omega_{k}dt)\\ \theta_{k-1}+\Omega_{k}dt \end{array}\right)$$

where *V_k_* and Ω*_k_* are the linear and angular velocities at the instant of time k.

### 3.2. Calibration Pattern Model

The calibration pattern, attached to the robot, is modeled as a set of N three-dimensional points grouped in a matrix **Q** = [(**Q**^1^)*^T^*,…, (**Q***^j^*)*^T^*,…, (**Q***^N^*)*^T^*]*^T^*, described in the pattern frame *O_P_*, where **Q***^j^* = [*x^j^, y^j^, z^j^*]*^T^* is the j-th point in the calibration pattern. It is important to mention that all of the N points are on the same plane, defined by the *x* and *y*-axis, assuming the *z*-axis orthogonal to that plane. In order to simplify calculations, we consider that the calibration pattern is at plane *z* = 0; thus, **Q***^j^* = [*x^j^,y^j^*, 0]*^T^*.

As the reference frame attached to the calibration pattern does not necessarily coincide with the robot frame, it is important to define a rotation RPR and translation TPR that describe the coordinate transformation between the pattern reference frame and the robot frame. After changing coordinates to the robot frame, the j-th point on the calibration pattern is represented as M*^j^*.

Because the pattern is fixed on a rigid structure, which ensures that its points are time invariant, we decided not to use the temporal sub-indexes *k* in the pattern points representation.

### 3.3. Camera Model

To use cameras for 3D reconstruction or object localization, it is necessary to establish the relationship between the 3D coordinates of a point in the world frame and its projection on the image plane, represented by 2D coordinates.

To define this relationship, the parameters for the camera calibration must be calculated. These parameters are divided in two groups: the intrinsic parameters, which transform a 3D point represented in the camera frame, *O_C_*, to a 2D point with pixel coordinates on the image plane; and the extrinsic parameters, that define a rigid-body transformation, RwC and TwC that transform a point in the global frame, *O_w_*, to the camera frame.

Based on the pinhole camera model [30], the intrinsic parameters of the camera can be arranged in a matrix **A**, in which we considered no radial distortion and no skew factor between the two axis of the image plane:

(3) $$\text{A}=\left(\begin{array}{lll} f_{u} & 0 & u_{0}\\ 0 & f_{v} & \nu_{0}\\ 0 & 0 & 1 \end{array}\right)$$

where *f_u_* and *f_v_* are the focal lengths in pixels for the u and v axis, and *u*_0_ and *v*_0_ are the pixel coordinates for the principal point in the image. The extrinsic parameters are the translation vector, TwC, in mm, and the rotation matrix RwC, which may be represented by a vector ωwC according to Rodrigues's formula [36]. Since there is more than one camera in the intelligent space, we use an index *i* to indicate the set of the *i*-th camera calibration parameters. Without loss of formalization, because the parameters will be frequently mentioned in this paper, the *C* and *w* indexes that represent the coordinate transformation from the global frame to the camera frame will be omitted to facilitate the equation reading. Thus, the extrinsic parameters of the *i*-th camera in the intelligent space will be represented as **T***^i^*, **R***^i^* and ω*^i^* and may be joined with the intrinsic parameters in a vector:

(4) $${\text{P}}^{i}={\left(f_{u}^{i},f_{v}^{i},u_{0}^{i},v_{0}^{i},{\left({\text{ω}}^{i}\right)}^{T},{\left({\text{T}}^{i}\right)}^{T}\right)}^{T}$$

In this work, a point on the calibration pattern represented as **M***^j^* = (*x^j^*, *y^j^*, *z^j^*)*^T^* is projected on the image plane as a two-dimensional point **m***^j^* = (*u^j^*, *v^j^*)*^T^*.

Therefore, for each camera in the intelligent space, we adopted the geometric projection model ${\text{m}}_{k}^{i,j}=h({\text{M}}^{j},{\text{X}}_{k},{\text{P}}^{i})$ to relate the three-dimensional point **M***^j^* on the calibration pattern, represented in the robot frame, to the point ${\text{m}}_{k}^{i,j}$ on the image plane of the camera *i* when the robot is in pose **X***_k_*. The function h is given by:

(5) $$\begin{array}{ll} {\text{m}}_{k}^{i,j} & =\left(\begin{array}{c} u_{k}^{i,j}\\ \nu_{k}^{i,j} \end{array}\right)\\ & =h({\text{M}}^{j},{\text{X}}_{k},{\text{P}}^{i}\\ & =\lambda_{k}^{i,j}{\text{A}}^{i}({\text{R}}^{i}{(}_{R}^{\omega }{\text{R}}_{k}{\text{M}}^{j}{+}_{R}^{\omega }{\text{T}}_{k})+{\text{T}}^{i}) \end{array}$$

where **A***^i^*, **R***^i^*, **T***^i^* are the matrices for the calibration parameters of camera *i*, $\lambda_{k}^{i,j}$ is a projective scale factor and RkRω and TRωk compose the rigid-body transformation that takes a point in the robot frame to the global frame. The rotation RkRω and translation TRωk are obtained from the robot pose **X***_k_*, as:

(6) RRωk=(cos(θk)sin(θk)0−sin(θk)cos(θk)0001)TRωk=(xkyk0)

Therefore, for a point **M***^j^* on the calibration pattern, with the robot located at **X***_k_* and a vector of parameters **P***^i^* for the camera *i*, the resulting non-homogeneous transformation *h* for the conventional pinhole camera model is defined as:

(7) $$h({\text{M}}^{j},{\text{X}}_{k},{\text{P}}^{i})=\left(\begin{array}{l} f_{u}^{i}\frac{R_{1,1}^{i}x_{k}^{j}+R_{1,2}^{i}y_{k}^{j}+R_{1,3}^{i}z_{k}^{j}+T_{1}^{i}}{R_{3,1}^{i}x_{k}^{j}+R_{3,2}^{i}y_{k}^{j}+R_{3,3}^{i}z_{k}^{j}+T_{3}^{i}}+u_{0}^{i}\\ f_{v}^{i}\frac{R_{2,1}^{i}x_{k}^{j}+R_{2,2}^{i}y_{k}^{j}+R_{2,3}^{i}z_{k}^{j}+T_{2}^{i}}{R_{3,1}^{1}x_{k}^{j}+R_{3,2}^{i}y_{k}^{j}+R_{3,3}^{i}z_{k}^{j}+T_{3}^{i}}+v_{0}^{i} \end{array}\right)$$

where $T_{l}^{i}$ represents the element at position *l* in the vector **T***^i^*, $R_{l,c}^{i}$ is the element at (*l*, *c*) in the rotation matrix **R***_i_*, represented by the vector ω*^i^* and $\left(x_{k}^{j},y_{k}^{j},z_{k}^{j}\right)$ are the coordinates of the point M*^j^* on the calibration pattern, when the robot is at X*_k_*, obtained from:

(8) $$\left(\begin{array}{c} x_{k}^{j}\\ y_{k}^{j}\\ z_{k}^{j} \end{array}\right){=}_{R}^{\omega }{\text{R}}_{k}{\text{M}}^{j}{+}_{R}^{\omega }{\text{T}}_{k}$$

Each one of the C cameras has a different calibration vector **P***^i^* that contains their parameters. The column vector **P** = ((**P**^1^)*^T^*,…, (**P***^i^*)*^T^*, …, (**P***^C^*)*^T^*)*^T^* includes all of the calibration vectors for all of the C cameras, representing the intelligent space complete calibration.

The set of measurements taken by camera *i* for all points on the calibration pattern, during a full sequence of robot motion, is given by **Y***^i^*, *i.e.*,

(9) $${\text{Y}}^{i}={\left({\left(m_{0}^{i,1}\right)}^{T},\ldots ,{\left(m_{K}^{i,N}\right)}^{T}\right)}^{T}$$

In the same way, the vector **Y** is defined by joining all measurements performed by all cameras, such as:

(10) $$\text{Y}={\left({\left({\text{Y}}^{1}\right)}^{T},\ldots ,{\left({\text{Y}}^{C}\right)}^{T}\right)}^{T}$$

From the function *h*, the mathematical relationship h involving all of the cameras calibration parameters **P**, the calibration pattern points **M**, the robot poses on the performed path **X** and the set of measurements **Y** can be defined as follows:

(11) Y=h(M,X,P)

## 4. Multi-Camera Network Calibration and Robot Localization

For the algorithm proposed in this work, the initial estimate of the camera calibration parameters is done based on a widely known algorithm [29].

After that, such parameters are improved using the robot's odometry data applying a maximum *a posteriori* (MAP) criterion. Therefore, the idea is to obtain a vector of parameters, represented as **Φ** and composed of the robot's pose vector and the calibration parameters that one wants to optimize, *i.e.*, **Φ** = (**P***^T^*, **X***^T^*). The MAP criterion is based on finding the maximum of:

(12) $$\Phi_{\text{MAP}}=\underset{\text{Φ}}{\text{max}}(p(\text{Y}|\text{Φ})p(\text{Φ}))$$

where *p*(**Y**|**Φ**) is a probability density function of the measurements **Y**, given the parameters **Φ**, and the function *p*(**Φ**) is a probability density function *a priori* of the parameters **Φ**.

For calculating the probability density function of the parameters **Φ**, we suppose that **X** and **P** are statistically independent. Thus, the distribution *a priori p*(**Φ**) can be obtained as *p*(**Φ**) = *p*(**X**)*p*(**P**).

The probability density function *a priori p*(**X**) is modeled as a Gaussian distribution with covariance matrix ∑*_X_* and a mean vector equal to the measurements obtained from the robot odometry **X̂** = **X***_odometry_*. The probability density function *a priori, p*(**P**), of the calibration parameters **P** is modeled as a Gaussian distribution with mean **P̂** equal to the initial values estimated with Zhang's algorithm [29].

Now, the function *p*(**Y**|Φ) is defined as a Gaussian distribution, where the mean is given by **Ŷ**=h(Φ) and the covariance matrix ∑*_Y_*, block diagonal, defines the uncertainty on the coordinates belonging to the points on the calibration pattern [37].

The MAP criterion of Equation (12) is equivalent to the following cost function, obtained by applying logarithms and eliminating the constants in the equation that do not change the minimum position:

(13) $$\begin{array}{c} {\text{Φ}}_{\text{MAP}}=\underset{\text{Φ}}{\text{min}}[{(\text{Y}-\text{h}(\text{Φ}))}^{T}\sum_{Y}^{-1}(\text{Y}-\text{h}(\Phi )+\\ {\left(\text{X}-\hat{\text{X}}\right)}^{T}\sum_{X}^{-1}\left(\text{X}-\hat{\text{X}}\right)+\\ {\left(\text{P}-\hat{\text{P}}\right)}^{T}\sum_{P}^{-1}\left(\text{P}-\hat{\text{P}}\right)] \end{array}$$

If the behavior of the distribution *a priori p*(**P**), represented by the covariance matrix ∑**_P_**, is considered unknown and, consequently, assuming that ‖∑**_P_**‖∞ is sufficiently large, then we can eliminate the latter term of Equation (13). Thus, the cost function for the parameters optimization is simplified to:

(14) $$\begin{array}{c} \Phi_{\text{MAP}}=\underset{\Phi }{\text{min}}[{(\text{Y}-\text{h}(\text{Φ}))}^{T}\sum_{Y}^{-1}\left(Y-\text{h}(\text{Φ}\right)+\\ {\left(\text{X}-\hat{\text{X}}\right)}^{T}\sum_{X}^{-1}\left(\text{X}-\hat{\text{X}}\right)] \end{array}$$

The cost functions given by Equations (13) and (14) can be minimized using an iterative optimization method. In this paper, the method chosen is the Levenberg-Marquardt method. It is important to notice that, in general, such cost functions have a very characteristic structure that can be used to decrease the complexity of each iteration [30].

With the MAP method proposed by Equation (14), it is possible to model the existing uncertainty in the odometry estimation. The distribution *p*(**X**) serves to “penalize” the robot poses, obtained from the odometry readings, which present larger uncertainties while forcing the algorithm to approximate the solution of the poses with less uncertainty. As a result, the proposed algorithm calculates more accurate calibration parameters at the same time that it localizes the robot in the workspace, considerably decreasing the odometry errors.

## 5. Results and Discussion

In order to validate the multi-camera structure of the intelligent space and the calibration algorithm proposed in this work, simulations and experiments were conducted, which are described in the following subsections. The objective of the simulation was to validate the proposed algorithm performance under different conditions. The experiments aim to analyze the hardware and software performance in the intelligent apace and the proposed algorithm for calibration and localization, described in Sections 2 and 4.

As mentioned before, in this work, the experiments were conducted in just one of the labs, with four cameras having some overlapping views. Yet, the way the proposed algorithm was implemented should allow its operation with non-overlapping cameras. However, experiments, including the entire group of 11 cameras, represent some of the future steps of this work and will not be presented in this paper.

### 5.1. Simulations

**Figure 9.**
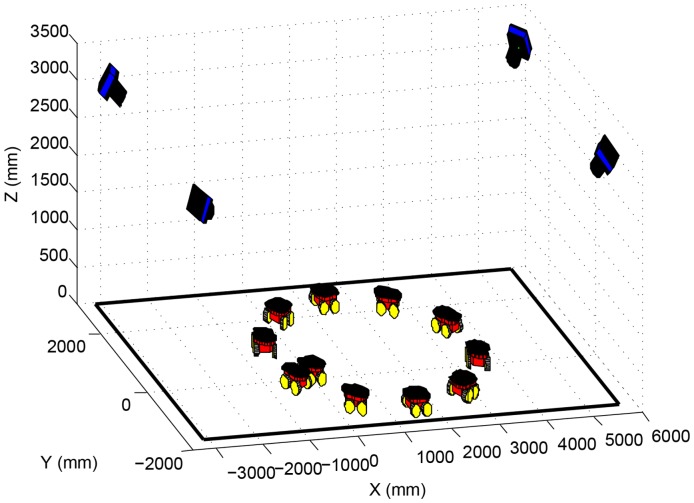
Simulated cameras and robot path in the laboratory.

In the simulations presented in this section, four cameras and one robot were simulated, as shown in Figure 9. The robot performed a circular path in the laboratory, as if it were controlled by a joystick, going through the four cameras' field of view. A 60 cm × 60 cm calibration pattern was considered fixed on top of the robot, with 21 equally distant marks, distributed in three lines.

The simulations aimed to analyze the proposed algorithm performance regarding the amount of robot poses used for calibration. Thus, the number of poses was varied from 10 to 100, with a step of five. It is important to notice that, for some poses, not every camera was able to see the entire robot. For example, if 10 poses were used in a simulation, the number of images captured by one of the cameras, showing the whole calibration pattern, could be less than 10. During the simulation, 120 experiments were made for every number of poses considered. Once from 10 to 100, with a step of five, results in 19 different setups, and considering 120 experiments for each setup, a total of 2280 experiments were made for this analysis.

**Figure 10.**
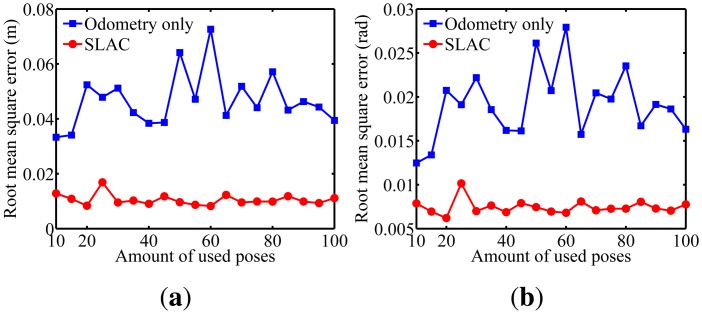
Robot's pose for different amounts of used poses. We can notice that both the position and orientation of the robot were improved with the proposed algorithm.

**Figure 11.**
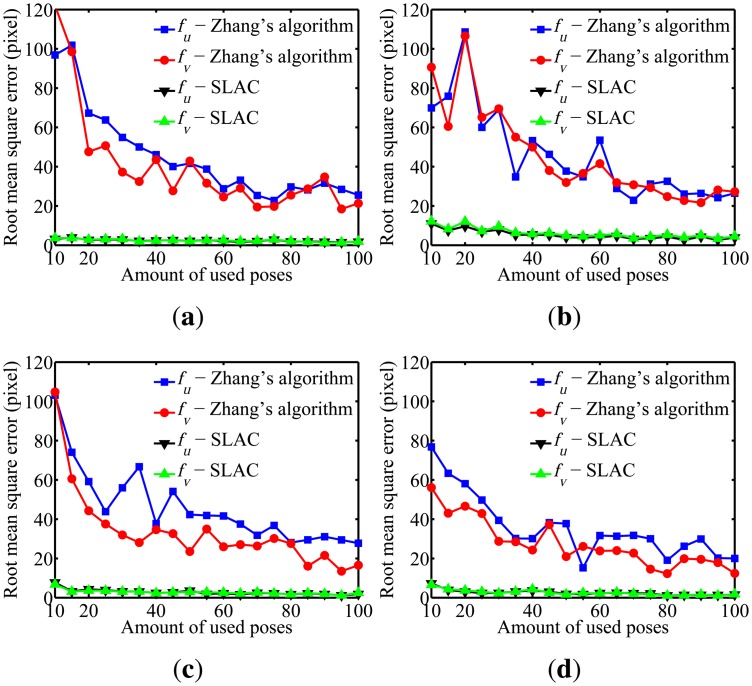
Simulation results for the *f_u_* and *f_v_* parameters for different amounts of used poses. (**a**) Camera 1; (**b**) Camera 2; (**c**) Camera 3; (**d**) Camera 4.

**Figure 12.**
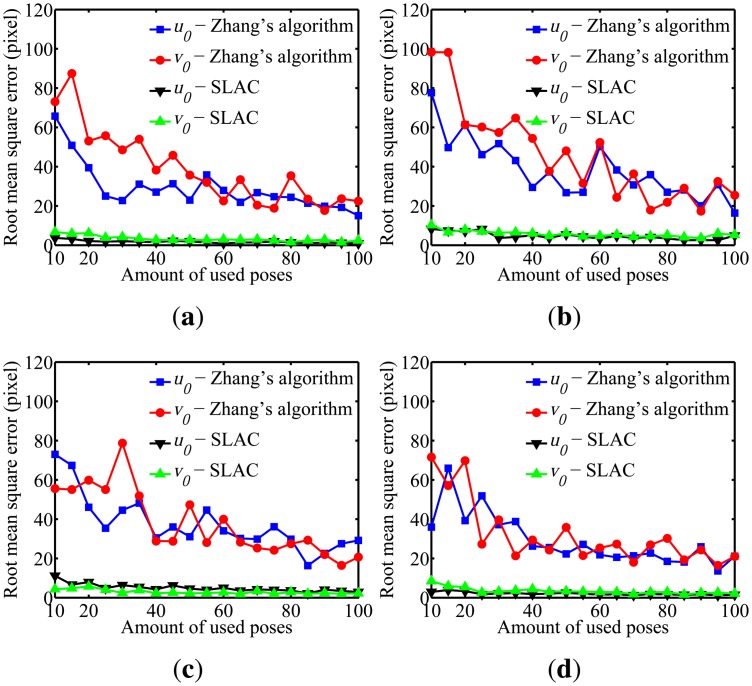
Simulation results for the u_0_ and v_0_ parameters for different amounts of used poses. (**a**) Camera 1; (**b**) Camera 2; (**c**) Camera 3; (**d**) Camera 4.

**Figure 13.**
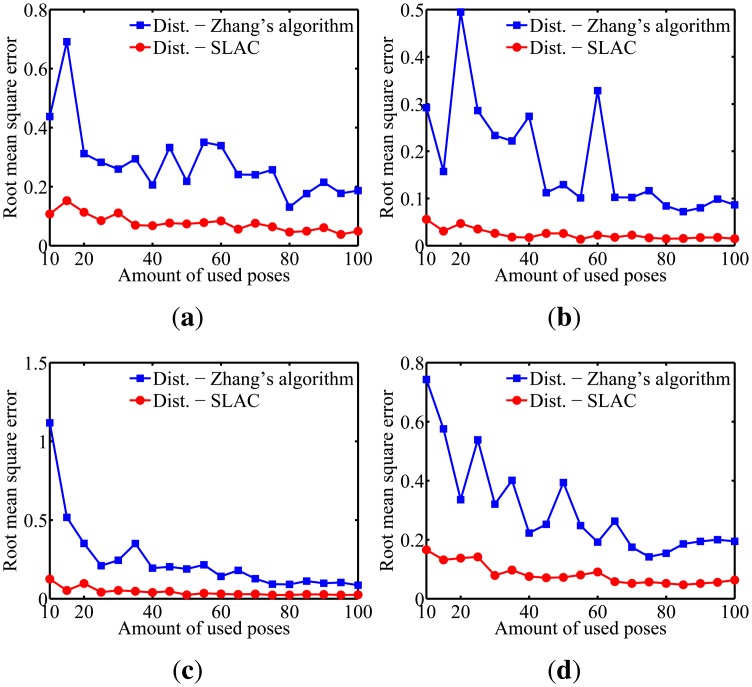
Simulation results for the radial distortion parameters for different amounts of used poses. (**a**) Camera 1; (**b**) Camera 2; (**c**) Camera 3; (**d**) Camera 4.

**Figure 14.**
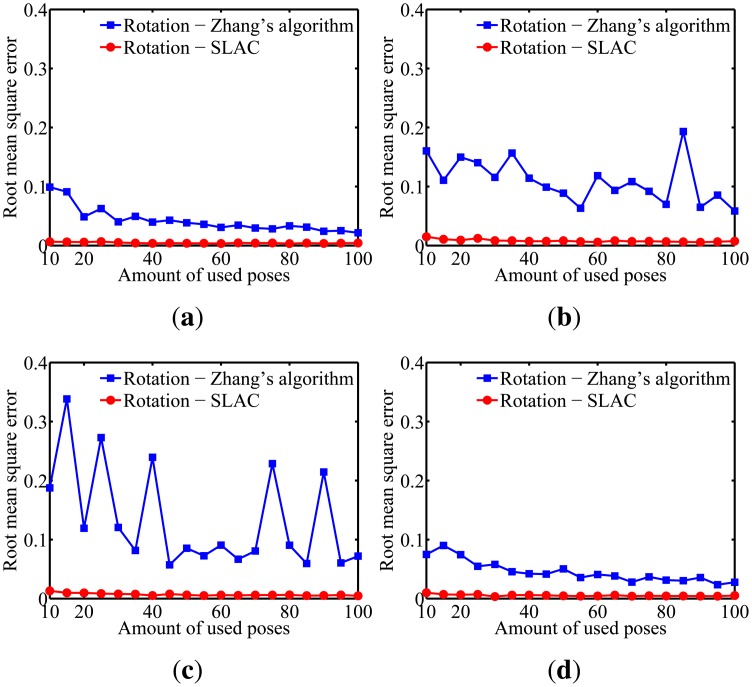
Simulation results for the rotation parameters for different amounts of used poses. (**a**) Camera 1; (**b**) Camera 2; (**c**) Camera 3; (**d**) Camera 4.

**Figure 15.**
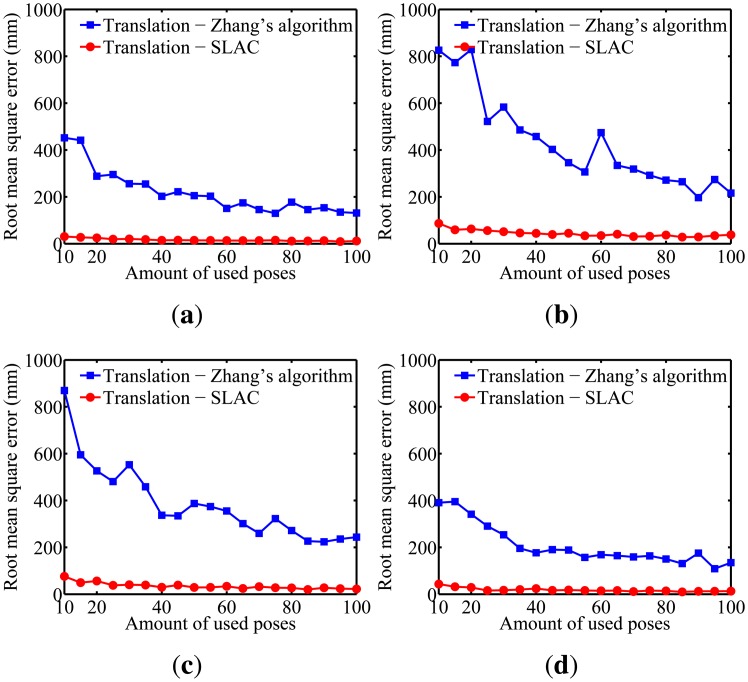
Simulation results for the translation parameters for different amounts of used poses. (**a**) Camera 1; (**b**) Camera 2; (**c**) Camera 3; (**d**) Camera 4.

To evaluate the performance of the proposed algorithm for localizing the robot, the mean squared error (RMSE) in the robot poses estimated by the odometry sensors and by the proposed algorithm are compared in Figure 10. Furthermore, the evaluation of the results on cameras calibration obtained by the proposed algorithm was done through comparing it to the well known Zhang algorithm [29]. Figures 11, 12, 13, 14 and 15 show the RMSE for the cameras' parameters estimated by our algorithm, noted in the graphs as SLAC (simultaneous localization and calibration) and Zhang's algorithm.

In order to simulate the robot odometry, a Gaussian noise was introduced with *μ* = 0 and *σ* = 10% of the robot's linear speed. Furthermore, a Gaussian noise with *μ* = 0 and *σ* = 10% of the robot's angular speed was added. To the image measurements m, a Gaussian noise with *μ* = 0.0 and *σ* = 1.0 pixels was considered. All of these noise setups are higher than the values measured in real experiments in our laboratory.

The graphs show that, for 20 or more robot poses, the proposed algorithm achieves a good performance. The error peak happened when 15 poses were used in the simulations. It is worth mentioning that the initial values of the calibration parameters, used to start the optimization done by the proposed algorithm, are very important. They may improve considerably the optimization and, thus, make the SLAC algorithm achieve better results. Besides that, by the graph shown in Figure 10, it is possible to conclude that the proposed algorithm increases the precision in localizing the robot by using visual information in the process.

### 5.2. Real Experiments

The real experiments were divided into two parts, both performed in the 40 m^2^ laboratory, using the four cameras. The 50 cm × 60 cm calibration pattern was fixed on the robot, as shown in Figure 2. As mentioned before, the calibration pattern is compounded by 24 marks, arranged in a 6 × 4 grid. At least 50 different robot poses were used in the real experiments, once simulations have demonstrated that the number of poses should be higher than 20.

**Figure 16.**
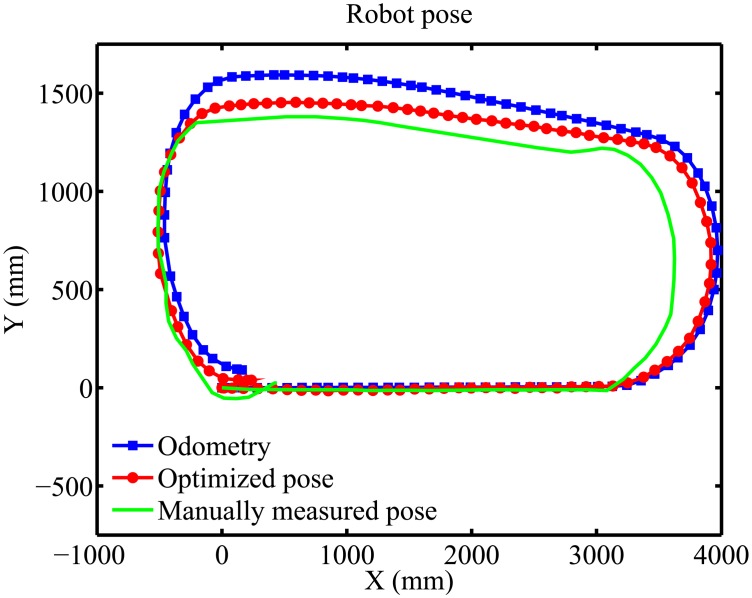
Experimental result for the robot's pose.

In the first experiment, the robot performed an oval path, going through the entire laboratory. The result for robot localization using the proposed algorithm is shown in Figure 16, and the results for the calibration parameters of each camera are shown in Table 1.

In order to evaluate the results for robot localization, the path performed by the mobile robot was measured manually, so it could be used as a reference. This reference path was obtained by attaching to the center of the robot a marker that drew down on the floor the trajectory during robot motion. After that, points on the path were measured considering a maximum error of 1.0 cm, that corresponds to the marker width. In Figure 16, the location of the robot given by its odometry and the proposed algorithm are compared to the reference path. It is possible to see that the robot poses estimated by our algorithm approximate the reference measurements.

**Figure 17.**
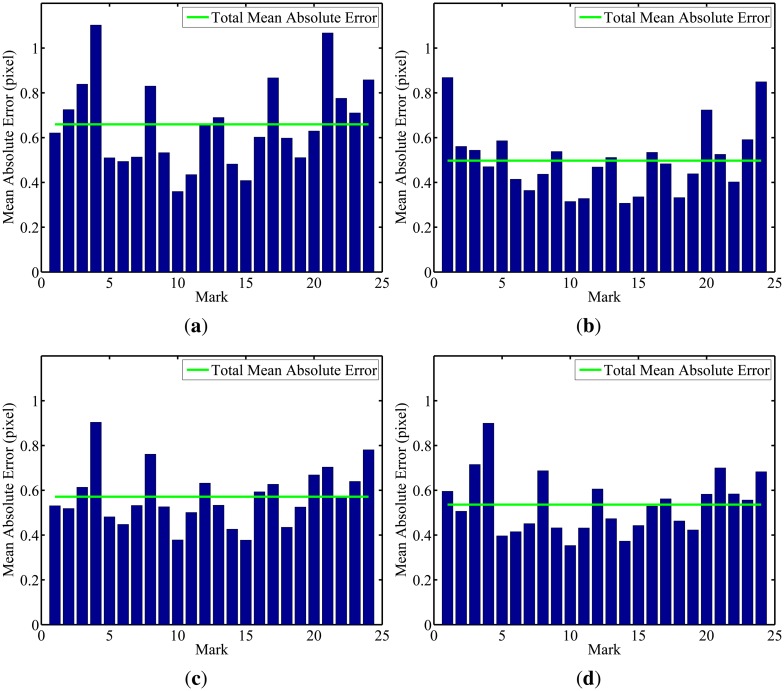
Reprojection error for the oval path. (**a**) Camera 1; (**b**) Camera 2; (**c**) Camera 3; (**d**) Camera 4.

**Figure 18.**
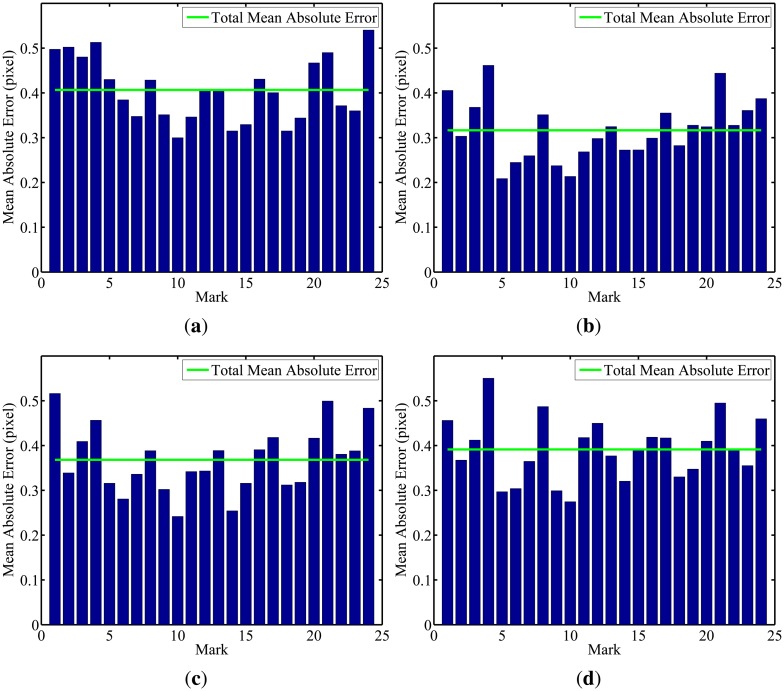
Reprojection error for the eight-shaped pathway. (**a**) Camera 1; (**b**) Camera 2; (**c**) Camera 3; (**d**) Camera 4.

In the second part of the experiment, the robot performed two different paths, an oval one and an eight-shaped one (lemniscate curve), once again covering the entire laboratory. In both cases, the four cameras in the intelligent space were used. To validate the calibration algorithm, reprojections were made using the calculated parameters and the calibration pattern images used in the experiment. The mean errors in the reprojections of the 3D points for each of the 24 marks is shown in Figures 17 and 18.

It is possible to notice by looking at the graphs that, in both experiments and for every camera, the mean error in the reprojections was smaller than 0.7 pixels. Such a low reprojection error attests the algorithm efficiency to camera calibration and, also, to robot localization.

## 6. Conclusions

This paper describes the intelligent space built at the Federal University of Espirito Santo. The objective is to localize and control mobile robots or robotic wheelchairs in order to help people. The intelligent space has 11 cameras distributed in two laboratories and the corridor between them. The cameras are fixed in the environment, and the image capturing is done synchronously.

Hardware and software architectures were developed to make the intelligent space work properly. The system is a client/server with TCP/IP connections, where the client coordinates the whole activity of the intelligent space and the servers provide the information needed for that. Each camera and robot is connected to a server, which is responsible for collecting the data from the sensors, processing them and sending the results to the client. Then, the client analyzes the data and determines which tasks would be performed. A communication protocol was defined, and the synchronization of the system was achieved satisfactorily.

Once the intelligent space cameras are used in 3D localizing of mobile robots and wheelchairs, it is necessary that they be properly calibrated. Therefore, a calibration method for a multi-camera network was presented in this paper.

Initially, several simulations were performed, and the results validated the proposed method, demonstrating the good performance of the algorithm in both calibration and localization. Real experiments in the intelligent space were also done and demonstrated the proper functioning of the system, validating both the architecture and the multi-camera calibration algorithm proposed. These results are encouraging and motivate us toward more sophisticated tests.

Future work includes controlling a mobile robot in the intelligent space without the need of many sensors onboard the robot. The robot will only have the basic sensors in order to perform essential actions, such as low-level control for linear and angular velocities and obstacle avoidance, since these tasks do not need to be coordinated by the intelligent space. Robot localization will be done using images captured by the cameras, and the robot pose will be recovered based on the parameters obtained by the calibration algorithm proposed. Once a mobile robot is controlled in the intelligent space, other tests will be conducted to also control a robotic wheelchair.

Besides that, another future intention is to test the calibration algorithm with the complete set of cameras with non-overlapping field of view. Furthermore, adjustments will be proposed to get the multi-camera calibration algorithm working on-line.

## Figures and Tables

**Table 1.** Calibrated parameters of the intelligent space's cameras

**Parameter**	**Camera 1**	**Camera 2**	**Camera 3 Camera 4**	
*f_u_*	747.4	685.8	683.7	697.7
*f_v_*	745.0	694.4	700.0	700.9
*u*_0_	555.1	487.0	184.0	541.8
*v*_0_	163.5	435.1	325.0	355.4
*k*_1_	−0.60	−0.30	−0.30	−0.29
*k*_2_	0.60	0.14	0.10	0.13

ω	1.59	2.05	1.07	1.17
	−1.29	1.26	2.45	− 2.41
	0.82	−0.329	−1.21	1.21

T (*mm*)	−560.3	−2,566	3,113	−353.4
	1,719	−2,713	−2,086	−175.0
	3,936	6,143	6,565	4,778
